# Costing the outpatient rehabilitation services: time-driven activity-based costing approach

**DOI:** 10.1186/s12962-022-00366-z

**Published:** 2022-07-14

**Authors:** Farzaneh Mohammadpour, Mehdi Basakha, Seyed Hossein Mohaqeqi Kamal, Nadia Azari

**Affiliations:** 1 Student Research Community, University of Social Welfare and Rehabilitation Sciences, Tehran, Iran; 2 Department of Social Welfare Management, School of Education Sciences and Social Welfare, Social Determinants of Health Research Center, University of Social Welfare and Rehabilitation Sciences, Tehran, 1985713834 Iran; 3 Department of Social Welfare Management, School of Education Sciences and Social Welfare, Social Welfare Management Research Center, University of Social Welfare and Rehabilitation Sciences, Tehran, Iran; 4 University of Social Welfare and Rehabilitation Sciences, Pediatric Neurorehabilitation Research Center, Tehran, Iran

**Keywords:** Time-driven activity based costing, Rehabilitation services, Costing, Outpatient services

## Abstract

**Background:**

Considering the importance of healthcare services costing in resource allocation, the aim of this study is to calculate the cost of rehabilitation services in an outpatient rehabilitation clinic in Tehran, Iran.

**Methods:**

The data for this study were categorised as financial data and information about the process of rehabilitation services. The first category was extracted from the financial documents and the second was obtained by observation of patient flow and interviews with the clinic staff in 2016. The cost of rehabilitation services has been estimated using the time-driven activity-based costing approach.

**Results:**

The findings show that the cost of physical occupational therapy in the Asma rehabilitation center was $18.79 per unit of service. This amount for speech therapy services was $17.23 to $19.40, taking into account the difference in the quality of the service delivered. The cost of mental health occupational therapy service was between $19.46 and $23.57. Comparing the cost of these services with the government’s tariffs makes it clear that there is a huge gap.

**Conclusion:**

The limited number of patients referred to the center makes the cost of one unit of rehabilitation services much higher than the official tariffs. This is true for almost all similar institutions and makes the profitability of small rehabilitation institutions extremely unstable. Therefore, proper marketing for rehabilitation services by promoting patient referral links with larger healthcare centers and reallocation of resources to the formation of integrated rehabilitation complexes can play a significant role in their profitability.

## Background

One of the main challenges in the sustainability of healthcare providers is the development of a cost information system that is suitable for pricing decisions, strategic management and resources allocation [1, 2]. Healthcare managers should be aware of all feasible costing methods to provide affordable and high-quality services as organisations face increasing diversity and complexity of services as well as budgetary limits [3]. Awareness of the cost structure through utilisation of an effective costing framework is essential for healthcare providers in order to survive in a competitive economic environment as well as for policy makers for healthcare resources allocation [4–6].

Different costing methods have been used for healthcare services, and over time, the accuracy of these methods have been enhanced. The traditional approaches, where there are no solid connections between the activities and the amount of resources used, can be only be employed in cases with limited activities and confined costs [7]. Due to this problem, the Activity-Based Costing (ABC) method has been developed. Nevertheless, setting up an ABC system is time consuming and the system should be updated regularly, which would increase the cost of accounting considerably [2, 8]. The constraints of this approach have led to a move towards the Time Driven Activity-Based Costing (TDABC) approach. This method was developed by Kaplan and Anderson [9, 10] in order to help healthcare organisations to identify unused capacity and enable them to reduce the cost of services resulting in more efficient service delivery and profitable schemes [11, 12].

Due to the importance of human resources in providing healthcare, personnel costs have a significant share in the costs of these services [13]. Different healthcare costing methods attribute different shares to personnel costs. Some studies have identified personnel costs as the largest part of healthcare costs 58 [14–16], while other studies place a lower proportion of the total cost on personnel [17]. Regardless of these differences, it is agreed that the hidden costs of unused human resources could be one potential reason for unaffordable healthcare services in developing countries [15, 18]. Therefore, the present study has tried to calculate the more accurate costs of providing rehabilitation services and to determine the unused capacity of human resources with the aim of paving the way for the reallocation of resources in healthcare sector.

### Iranian healthcare and rehabilitation services profile

The Ministry of Health and Medical Education (MOHME) is responsible for public health in Iran. Healthcare services are accessible through a number of different public and private providers. A significant portion of health services in Iran are financed by the Ministry of Welfare and Social Security through specialised insurer organisations including Social Security Organisation, the Army Medical Insurance Organisation and Health Insurance Organisation [19]. The coverage rate of social insurance in Iran is high (more than 90% of Iranian people have insurance), but these insurance policies do not provide adequate financial coverage and out-of-pocket (OOP) payments to offset a high proportion of healthcare expenditure [20]. The OOP health payments have the largest contribution in healthcare financing in Iran, accounting for over 31.4% of total healthcare expenditure and 37.6% of rehabilitation services expenditure [21].

Despite the challenge of chronic disease in the present century, the increase in the elderly population and the significant relationship between the need for rehabilitation services and catastrophic expenditures [22–24], the role of rehabilitation services in the Iranian healthcare system is still underestimated and the government and social insurers have drastically reduced their spending in recent years [25]. This makes access to rehabilitation services more difficult for many poorer groups and exacerbates the social cost of disability. Regrettably, most rehabilitation services are not covered by social insurances resulting in reduced utilisation of services among these groups of people. Accordingly, direct financing and imposing healthcare expenditure on households has led to the spread of the catastrophic expenditure [26].

Rehabilitation service providers in Iran include public, private, and charitable organisations. Their tariffs are set annually by the MOHME. The Asma Rehabilitation Centre (ARC) is affiliated to the University of Social Welfare and Rehabilitation Sciences and is funded by the MOHME. Therefore, the ARC is an educational therapeutic center and provides internship opportunities for the students of rehabilitation sciences as well as providing rehabilitative services. The ARC has a range of specialised departments, including speech therapy, physical occupational therapy, mental health occupational therapy, psychology, audiology, neuro-feedback, hand occupational therapy, and physiotherapy.

The present study used TDABC [11, 27] to calculate the cost of speech therapy services, physical occupational therapy, and mental health occupational therapy services at the ARC. By calculating the unused capacity of each department, the management of the ARC is able to derive the appropriate decisions for developing a profitable business. This study intends to calculate the cost of most repeated services based on their contribution to the workload and earnings of the ARC. The implicit purpose of this study is to raise public awareness of the sustainability of rehabilitation services.

## Methods

There are two main approaches to healthcare costing; highly detailed bottom-up costing and top-down approach. If detailed information is available, the bottom-up approach is recommended [28]. However, access to this information is limited in public organisations in developing countries, including Iran. Inescapably, the present study uses top-downapproach in costing the selected rehabilitation services in the ARC. The top-down method is suitable for low-volume, low-cost procedures and it is based on average costs [28].

**Figure 1:**
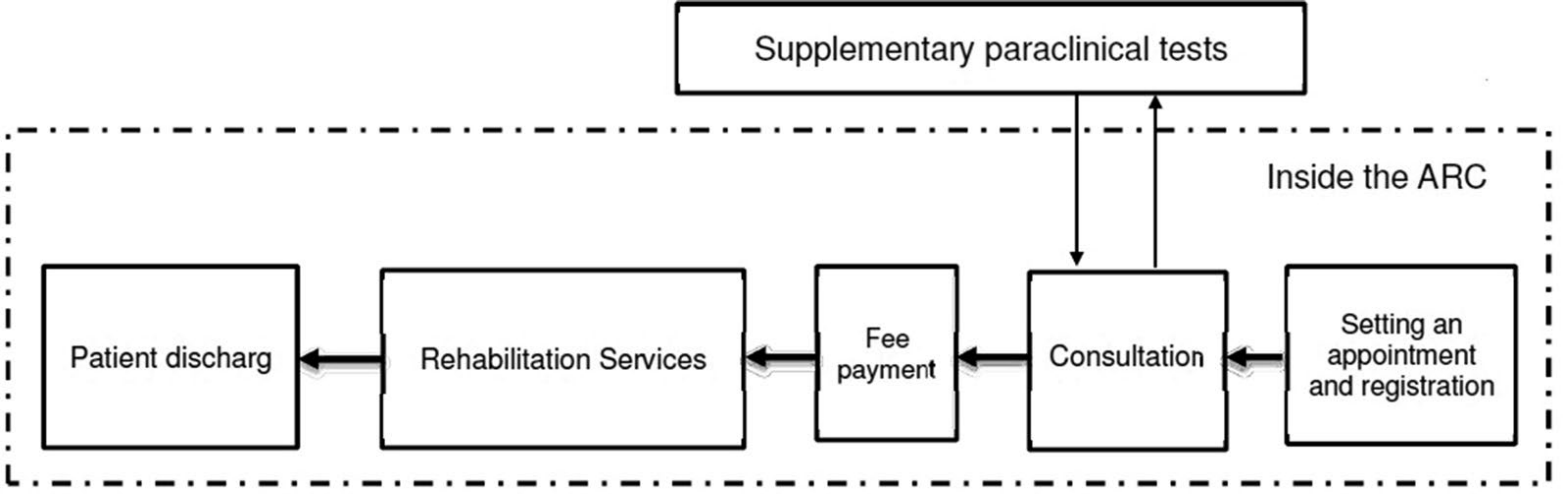
Outpatient rehabilitation services process map in ARC

The data needed for this research can be divided into two categories: financial data and information about the process of providing the rehabilitation services. The first category includes personnel costs, depreciation cost for office equipment and medical equipment, rent, and overhead costs. These were collected from the financial documents of the ARC in 2016. The second part of data was gathered by observing the services processes and by conducting informal interviews with the staff of the ARC after obtaining the necessary permissions. In TDABC method, estimation of two parameters are necessary: cost of a unit of resources, and the time required for each activity [17, 29]. More precisely, the TDABC process has been performed based on the following steps [27]:

1. Identifying the various departments: First, a map of the rehabilitation services process was drawn up by observing the process of activities in different departments and talking with the authorities in each department. Figure 1 represents the process map of these three services. Next, the various resource groups were identified.
2. Estimating the total cost of each resource group: The cost groups were identified through interviews with the Finance and Property Departments. Cost groups include buildings, overheads, human resources, and equipment (including medical equipment and office equipment). Through the aggregation of these costs, the total cost of each section was determined. Then, direct and indirect costs were calculated for each department. Costs that could not be categorised in the above categories (such as educational and research expenses, printing and purchasing of periodicals, the cost of materials and supplies, and the cost of transport and communications) were included as other overhead costs. The area (squared meter) occupied by each department was considered as basis of cost sharing for the building, and maintenance of other assets.
3. Estimating the practical capacity of resource groups: At this stage, the practical capacity of each group was calculated. Staff hours are from 8:00 am to 2:30 pm from Saturday to Wednesday, and from 8:00 am to 1:00 pm on Thursdays. Therefore, the total working time is 1956.5 h per year (117,390 min per year). Subsequently, the practical capacity of each group was considered to be 85% of the theoretical capacity (Kaplan and Anderson, 2007) equal to 99,781 min in a year. The practical capacity of medical and non-medical equipment and machinery was also considered in terms of the useful life of the equipment.
4. Calculating the unit cost of each resource group: After calculating the total cost and practical capacity for each cost group, the cost of each unit was obtained by dividing these two variables into each other.
5. Identifying the time equation: Once a patient has been referred to a unit, the time taken for them receive the service must be calculated. This time was measured by observation and timing for four patients, allowing time equations for rehabilitation services to be determined [2].
6. Aggregating the costs and calculating the final price of each service: This step was acceded by multiplying the unit cost of each resource group in time equations.

## Results

### The cost of rehabilitation services

The number of rehabilitation sessions required by each patient varies according to the severity of the disability and the type of service required. Sometimes, due to the financial hardship, the patient does not attend all the sessions. Based on the time equations, the total personnel costs of each department were obtained per unit of service (Table 1).

**Table 1:** Personnel cost for a regular 10-session rehabilitation

	Time (Minutes)	Cost per minute ($)		Cost ($)	
		MQ	HQ	MQ	HQ
Reception	1.52	0.08	0.08	0.12	0.12
Counseling	28	0.18	0.18	5.04	5.04
Speech therapy	31.3	0.11	0.18	3.44	5.63
Evaluation	12	0.18	0.18	2.16	2.16
Cashier	1.3	0.08	0.08	0.10	0.10
Total cost of a regular 10-session Speech Therapy				4	6.18
Reception	1.52	0.08	0.08	0.12	0.12
Counseling	28	0.18	0.18	5.04	5.04
Mental health occupational therapy	30	0.08	0.21	2.4	6.3
Evaluation	12	0.18	0.18	2.16	2.16
Cashier	1.3	0.08	0.08	0.10	0.10
Total cost of a regular 10-session Mental health occupational therapy				2.95	7.07
Reception	1.52	0.08	0.08	0.12	0.12
Counseling	28	0.18	0.18	5.04	5.04
Physical occupational therapy	53.6	0.09	-	48.24	-
Evaluation	12	0.18	0.18	2.16	2.16
Cashier	1.3	0.08	0.08	0.10	0.10
Total cost of a regular 10-session Physical occupational therapy				5.34	−

For departments where services are provided by a workforce with varying degrees of expertise (including speech therapy and mental health occupational therapy), rehabilitation activities were divided in terms of quality. High quality (HQ) services were defined as those delivered by rehabilitation specialists with a Ph.D. in a relevant field while medium quality (MQ) services were delivered by rehabilitation experts with a bachelor's or master’s degree in that field. By placing the cost per minute for the rehabilitation specialists (HQ) and rehabilitation (MQ) experts in to the time equations, the cost of HQ and MQ services can be calculated. In order to estimate the average rehabilitation session for each patient, we have considered a regular 10-session course of treatment for these services. Given that, the counseling and evaluation of the general practitioner are provided only twice, so this item is counted only at the beginning and the end of a regular 10-session rehabilitation.

**Table 2:** Cost of resources for rehabilitation services (US $)

	Speech Therapy	Mental Health Occupational Therapy	Physical occupational Therapy
Total non-personnel costs	18,663.57	17,145.43	10,032.19
Rent	8049.95	8017.94	4345.06
Utilities	442.51	440.76	238.84
Medical equipment depreciation	134.76	302.65	98.79
Office equipment depreciation	324.54	214.18	186.85
Outsourcing contract services	3661.70	2413.78	1911.31
Repair and Maintenance of equipment	58.59	38.61	30.58
Repair and maintenance of other assets	5248.75	5227.88	2833.06
Other overhead cost	742.77	489.63	387.70
Non-personnel cost of a rehabilitation session	8.37	10.43	9.56
Personnel cost of a MQ rehabilitation session	4.20	3.15	5.54
Personnel cost of a HQ rehabilitation session	6.37	7.26	-
Non-technical personnel cost	4.66	5.88	3.69
Total cost of a MQ rehabilitation session	17.23	19.46	18.79
Total cost of a HQ rehabilitation session	19.40	23.57	-

The numbers in parentheses represent the share of each cost in the total cost

The non-personnel costs of each department are also admeasured according to the number of visits or the area occupied by the department. For this purpose, the cost of depreciation of equipment, rent of the building, repair and maintenance costs, and administrative costs were calculated for the different departments according to the contribution of each department to the activities of the institute.

The cost of rehabilitation services, categorised by personnel and non-personnel costs, of each department is also presented in Table 2. The highest non-personnel costs in the physical occupational therapy and speech therapy sectors were related to the “other overhead cost”; while in the mental health occupational therapy department, the cost of renting was the highest and accounted for the highest proportion of non-personnel costs. Given the small scale of the ARC, the cost of repair and maintenance of office equipment has been the lowest non-personnel cost for all three departments. Of the total cost of a speech therapy, mental health occupational therapy, and physical occupational therapy session, 8.37 $, 10.43 $ and 9.56 $ were related to non-personnel costs, respectively.

**Table 3:** Profitability of a regular session for rehabilitation services ($)

Rehabilitation service	Tariffs	Cost		Gross profit (Lost)	
		MQ	HQ	MQ	HQ
Speech therapy	7.01	17.23	19.40	−10.22	−12.39
Mental health occupational therapy	6.17	19.46	23.57	−13.29	−17.40
Physical occupational therapy	7.85	18.79	−	−10.94	−

A closer look at the cost components shows that the shares of employee compensation and benefit in the total cost of speech therapy, physical occupational therapy, and mental health occupational therapy services were 4.20$, 3.15$ and 5.54$, respectively.

### Profitability of the rehabilitation services

The government’s tariffs for a session of speech therapy, physical occupational therapy, and mental health occupational therapy in 2017 are shown in Table 3. The government’s tariffs did not cover the entire costs of any of these services for the ARC. The comparison of the tariffs for rehabilitation services and their cost shows that it is clear that the ARC has suffered losses for the provision of the services in 2017. The gap in the delivery cost and government tariffs for some health services has been reported in various studies [30, 31]. The largest gross loss in the clinical activities was due to the high-quality health mental occupational therapy.

### Unused human capital capacity

The practical capacity of personnel was calculated throughout the year. By multiplying the practical capacity of an individual and the number of personnel in each department, the practical capacity of that department is specified.

The comparison of unused capacity shows that the clinic’s cashier has the highest rate of idle time (Table 4). Total cashier activities in the clinic account for only 14.2% of his practical capacity. This percentage was less than 17% for receptionists. Rehabilitation specialist staffs also have a high rate of unused capacity. Meanwhile, mental health occupational therapists have the highest work less time (83% of total practical capacity) among the different departments. Interestingly, general practitioners in the clinic are the group whose activity time and practical capacity are very similar.

**Table 4:** Different capacities for rehabilitation services (min)

	Practical capacity	Used capacity	Unused capacity
Secretary	117,390 (100%)	19,537 (16.64%)	97,853 (83.36%)
General practitioner	224,640 (100%)	161,364 (71.83%)	63,276 (28.17%)
Speech therapist	255,060 (100%)	69,799 (27.36%)	185,261 (72.63%)
Mental health occupational therapist	272,220 (100%)	44,100 (16.20%)	228,120 (83.80%)
Physical occupational therapist	234,780 (100%)	62,390 (26.57%)	172,390 (73.43%)
Cashier	117,390 (100%)	16,709 (14.23%)	100,681 (85.77%)

The numbers in parentheses represent the share of each capacity in practical capacity

The unused capacity of human resources in the ARC shows that a new combination of work reassignments and better management of human resources could lead to more efficient workforce utilisation and lower average cost of personnel. For example, if the clinic has plans to expand rehabilitation activities, given the unused capacity calculated, it only requires more general practitioners to be appointed. Therefore, the development of rehabilitation activities at the ARC not only does not have high costs, but also reduces per capita indirect costs and ultimately reduces the cost of the services.

## Discussion

The literature lacks case studies of TDABC application in different rehabilitation services settings [15], which makes the cost of rehabilitation services non-transparent for providers, social insurance organisations and regulatory entities. This lack of transparency challenges the pricing of these services and negatively affects resources productivity and healthcare service utilization. Following the purpose of the study, it was observed that the cost of providing the rehabilitation services at the ARC is much higher than the government’s tariffs for these services. Although the ARC is a public institution and its losses are offset by the government budget, it is very likely that other rehabilitation centers will be in a similar situation [14, 32, 33]. The calculations showed that the cost of high-quality services for speech therapy and mental health occupational therapy was 2.76 and 3.82 times of the tariffs of these services, respectively. This ratio was 2.39 for physical occupational therapy services. The highest share of the cost of providing rehabilitation services, especially high-quality services, is related to human resource costs. Among the various types of personnel costs, the compensation of the rehabilitation specialists has the highest share. Indeed, the reason for this high cost should be inquired in the high work less time of rehabilitation specialists.

The highest non-personnel costs are due to "other overhead costs”; the most important of which is the building leasing cost. Therefore, avoiding the leasing costs (through resources reallocation like purchasing a building or integration with other rehabilitation institutions) can play an important role in the profitability of the institution's services. The rent and cost of buildings depreciation in Iran is burdensome [16, 32], and so the provision of rehabilitation services in joint settings, avoiding geographical dispersion, can help to reduce service provider’s costs and make their services more profitable. This matter emphasises the importance of resource management and the production scale in the ARC. In other words, due to the low number of patients, overhead costs were divided among a few patients and this contributed significantly to the high cost of rehabilitation services. With that in mind, it is expected that an increase in the number of patients at the ARC could significantly reduce the unit cost of the rehabilitation services and make its activities profitable. This finding can be extended to all rehabilitation clinics that operate on a small scale.

Healthcare is one of the services that has a high diversity of human resources with cost variations of more than 10:1[29]. In this regard, the use of the TDABC in this sector could be effective in determining the exact contribution of each type of human resources to the total cost. Studies in Iran have often considered lump sum personnel costs regardless of the time required to provide health care services [32, 34], that is why the personnel cost makes up a large portion of total costs and makes health care services so expensive. Therefore, use of the TDABC method can be a remedy for controlling costs and price inflation in the health sector. Application of this method could improve human resources management for healthcare providers and ensure affordable rehabilitation services.

## Conclusion

In the present study, firstly, costs are calculated using a more accurate method based on the allocated time for each activity. Then, through mapping the process, all stages of the rehabilitation service have been identified for the ARC. This framework facilitates the identification of cost drivers of providing rehabilitation services at the ARC. The findings indicate a significant gap between the cost of services and government tariffs. One of the main reasons for this gap is the existence of unused human resource capacity in the ARC.

Thereupon, if the ARC’s staff operate in a situation where their utilised capacity increases, fixed costs per unit of service will be reduced and result in lower prices.

One consequence of the high cost of rehabilitation services at the ARC was the formation of the gap between the cost of service and government tariffs. As a result of this gap, the ARC activities have been financially detrimental. However, a more accurate costing framework may contribute to lower the costs and increase revenues for rehabilitation activities.

This study attempts to apply a more accurate method for costing rehabilitation services, in addition to sensitisation about the loss of rehabilitation activities for small institutions. Long-term losses for rehabilitation clinics will limit the future of such services and will put the welfare community at a greater disadvantage. This study aimed to show that the government tariffs for rehabilitation services are significantly lower than the costs of providing these services. Second, most of rehabilitation services are not covered by Iranian social insurances, and tariffs are not affordable for families in need of rehabilitation services. In addition to inspire advocacy of social insurance provision for low-income families, this study sought to suggest an improved costing method for rehabilitation providers. Evidently, TDABC method may provide a more realistic cost structure information and other valuable information, such as a realistic profit/lost overview, unused human capacity and a basis for improved resources management for health care providers. All of this may enhance the availability and accessibility of rehabilitation services.

The research has been accompanied by a few limitations. One of the limitations of the research was that not all rehabilitation activities cost has been calculated. This selection of activities was based on the number of patients referred to departments. Time and money constraints made it impossible for calculations to be made for all departments. In addition, it should be stated that all information was provided by the ARC and the accuracy of this information has not been independently verified. Another issue is the lack of links between the cost and value of rehabilitation services for patients. The actual value of the utility that rehabilitation services given to clients may be more or less than the estimated cost. Ultimately, these calculations were related to a small-scale rehabilitation center and the findings cannot be generalised to all active businesses in the rehabilitation services.

## Data Availability

The datasets generated and analysed during the current study are not publicly available due to the confidentiality of organizational data; but are available from the corresponding author on reasonable request.

## Abbreviations

ABC: Activity based costing
ARC: The Asma Rehabilitation Center
TDABC: Time-driver activity based costing
HQ: High quality service
MQ: Medium quality service
